# BaleUAVision: Hay Bales UAV Captured Dataset

**DOI:** 10.1038/s41597-026-06622-8

**Published:** 2026-01-29

**Authors:** Georgios D. Karatzinis, Socratis Gkelios, Athanasios Ch. Kapoutsis

**Affiliations:** Information Technologies Institute (ITI), Centre of Research and Technology Hellas (CERTH), Thessaloniki, Greece

**Keywords:** Agriculture, Environmental sciences, Databases

## Abstract

Efficient hay bale detection and counting are essential tasks within modern precision agriculture, significantly impacting yield estimation, logistics, and sustainable resource management. To address current limitations in dataset quality and environmental representation, we introduce *BaleUAVision*, a comprehensive dataset consisting of 2,599 high-resolution RGB images, each containing numerous human-annotated hay bales. Captured by Unmanned Aerial Vehicles (UAVs) across 16 diverse agricultural fields in Northern Greece, the dataset includes varying flight altitudes (50–100 meters), diverse speeds (3.7–5 m/s), and overlapping strategies to ensure robust data representation. *BaleUAVision* provides rich annotations through polygon-based semantic segmentation in multiple formats (COCO, CSV, JSON, YOLO, segmentation masks) and high-quality orthomosaics for precise spatial analysis. Technical validation demonstrated the dataset’s effectiveness in training robust hay bale detection models using YOLOv11, achieving high precision and recall under varying geographic and altitude conditions. Specifically, the dataset supported effective generalization across geographically distinct areas (Xanthi and Drama regions) and varying altitudes, highlighting its utility in real-world UAV operations. The dataset and supplementary tools, scripts, and analyses are publicly available on Zenodo and GitHub respectively, following FAIR principles to support wide-reaching applicability within the research community.

## Background & Summary

Efficient and automated hay bale management is vital for modern precision agriculture, as accurate detection and counting provide critical insights into yield estimation, logistics, and resource allocation^1^. By precisely tracking the number and location of bales, farmers can optimize harvesting operations, reduce labor requirements, and improve the overall use of resources^2^. High-resolution and timely information on bale distribution further aids in planning for collection, storage, and transportation—ensuring timely delivery, maintaining feed quality for livestock, and minimizing operational expenses^3^. In addition to logistics, tracking the distribution of the hay bales can serve as a proxy for field management techniques and environmental sustainability by revealing how frequently and intensively fields are harvested^4^. Taken together, these capabilities underscore the value of precision agriculture techniques, guiding efficient use of machinery, targeted interventions - such as optimizing bale pickup routes^5^ - and better monitoring of production in large areas.

Unmanned Aerial Vehicles (UAVs) have emerged as a valuable tool in precision agriculture, providing a versatile and cost-effective platform for obtaining high-resolution imagery (e.g.^6–8^). UAVs can collect data across broad areas, in changing weather conditions (within limits), and at user-defined revisit frequencies, making them excellent for crop monitoring, field condition assessment, and agricultural task automation (e.g.^9,10^). Recent research has shown that integrating computer vision and machine learning approaches to UAV-acquired imagery is useful for a variety of agricultural applications, such as crop categorization, weed detection, and yield estimation (e.g.^11,12^). However, using these algorithms for the detection and counting of hay bales is somewhat unexplored, especially with high-quality, diversified datasets.

Current hay bale detection methods often rely on manual labor or less sophisticated automated techniques, both of which are time-consuming, labor-intensive, and prone to inaccuracies. Progress in detection methods and, in general, the adoption of AI techniques in Precision Agriculture has been hindered by limitations in available datasets, which frequently suffer from low-resolution imagery, limited field conditions, and annotation disparities (e.g.^13,14^). For example, hay bale data sets such as those used in^15,16^ often exhibit limited altitude ranges, uniform flight parameters, and a lack of environmental diversity, preventing their applicability to real-world agricultural settings. Previous work frequently used static flight paths and fixed altitudes, failing to capture the natural variability in field layouts, crop types, and seasonal conditions. Furthermore, many studies have focused exclusively on individual object detection, neglecting the potential benefits of different flight mission characteristics, orthomosaic generation, and segmentation-based annotations, all of which are quite crucial for developing robust machine learning models. While datasets like HarvestNet^17^ address some aspects of agricultural monitoring by focusing on smallholder farming activity, their dependence on satellite imagery usually results in low-resolution images, limited temporal resolution, and environmental inconsistencies, rendering accurate bale detection and counting a challenging task. These constraints highlight the need for a much-enhanced dataset—a large-scale, high-quality resource that includes different flight characteristics. Such a dataset would allow for the development of more robust and accurate detection models, so a new data collection campaign is required to create a more representative dataset that captures a broader range of environmental conditions, higher-resolution imagery, and more comprehensive annotations, ultimately advancing automated hay bale detection and resource management in precision agriculture.

The Segment Anything Model (SAM)^18^, developed by Meta AI, represents a milestone in image segmentation due to its unprecedented generalization capabilities across diverse visual domains, enabling accurate zero-shot predictions without requiring additional training on new objects. Building on this foundation, recent research has explored its utility for geospatial contexts, where segmentation is a critical task for monitoring land cover, urban growth, and environmental change. In particular, a study demonstrated that SAM’s zero- and one-shot learning capabilities can significantly reduce the annotation burden in remote sensing applications, highlighting its promise for multi-scale aerial and satellite imagery^19^. At the same time, this study also revealed challenges relevant to agricultural UAV tasks, such as difficulties in segmenting small or repetitive objects at coarser resolutions and a tendency to overestimate boundaries in heterogeneous vegetation environments. This dual perspective underscores both SAM’s foundational importance in computer vision and the need for domain-specific resources to ensure reliable performance in agriculture-focused applications.

Based on the existing literature, no published study has directly applied SAM for instance- or bale-level segmentation of tightly packed agricultural objects such as hay bales. However, several works in agricultural and remote sensing domains reveal SAM’s limitations under domain shift or challenging visual conditions. More specifically, one study^20^ shows that SAM’s performance deteriorates in images with unclear boundaries and low contrast, particularly in remote sensing of agricultural and urban green spaces. Another work^21^ finds that in crop disease and pest segmentation, SAM does not achieve satisfactory segmentation performance without domain adaptation and requires prompt corrections. In another recent paper^22^ it has been highlighted that in UAV forest floor imagery a challenge remains due to high natural variability, quickly changing environmental parameters, and ambiguous annotations due to unclear definitions and thus SAM must be fine-tuned to produce accurate masks rather than relying on zero-shot performance. Similarly, it has been reported that SAM achieves only about 58% correct boundary detection when delineating agricultural fields under zero-shot conditions^23^. These findings support our reasoning that a high-quality, domain-specific dataset is necessary to fine-tune or adapt SAM (or similar models) to work reliably in agricultural UAV settings, especially for precise, small-object instance segmentation tasks like hay bale detection, counting, field condition assessment and resource allocation optimization.

Beyond these limitations, *BaleUAVision* is designed to complement recent advances in general-purpose vision models. By providing dense, polygon-based annotations hay bales under diverse UAV conditions, the dataset enables targeted fine-tuning of foundation models such as SAM. This is particularly valuable for domain-specific edge cases (small, repetitive, and visually homogeneous agricultural objects), where zero-shot performance is insufficient. In this way, *BaleUAVision* not only supports the development of dedicated detection pipelines but also serves as a high-quality resource for adapting and evaluating large vision models in agricultural UAV tasks, particularly in settings where zero-shot performance is insufficient.

**Fig. 1:**
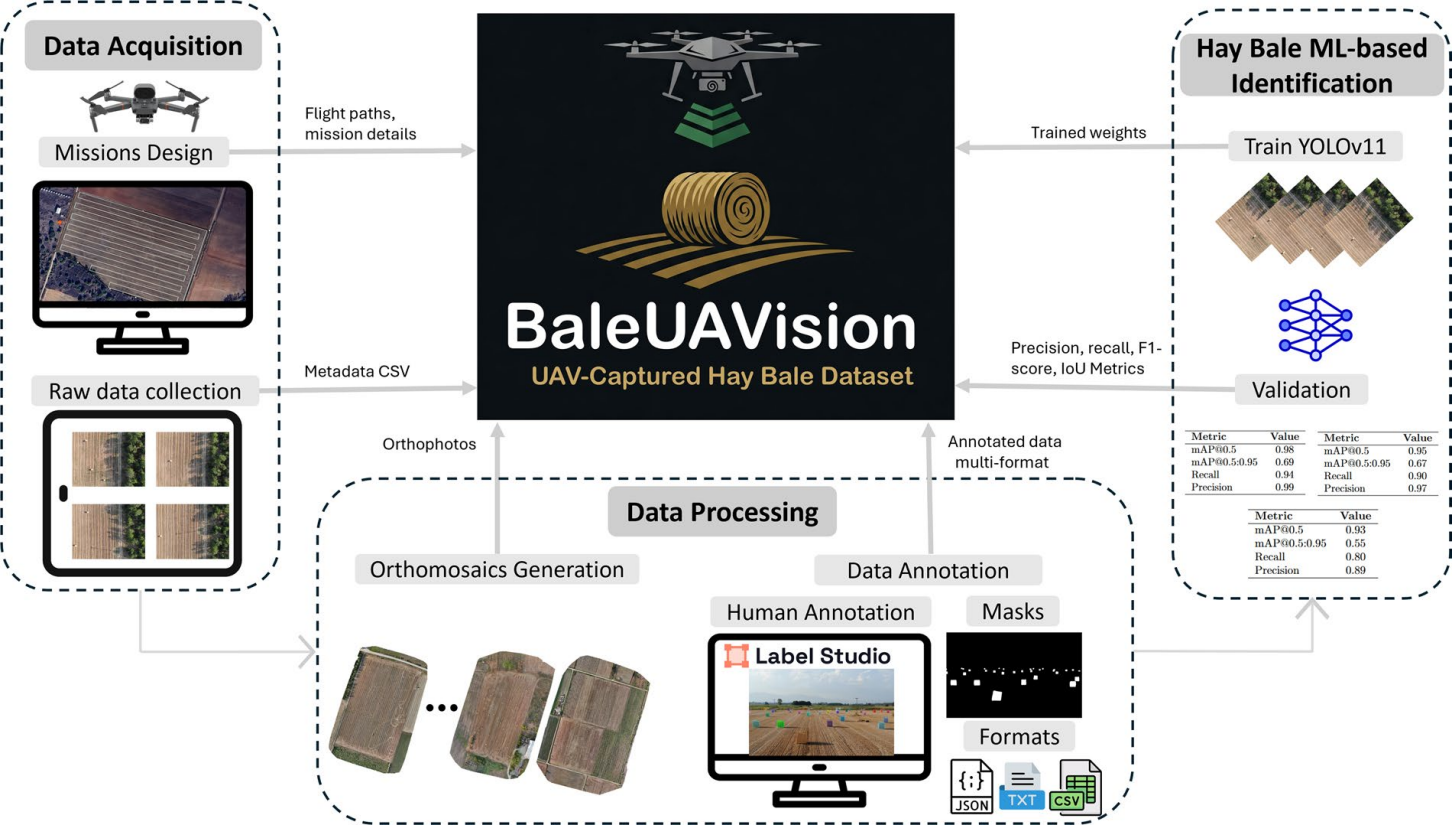
Workflow of the *BaleUAVision* dataset creation pipeline. The pipeline consists of three main steps: Data Acquisition, which involves UAV mission planning, raw image collection, and the recording of flight paths, mission details, and metadata CSVs; Data Processing, where high-resolution orthophotos and orthomosaics were generated using WebODM software, and semantic segmentation annotations were created using Label Studio; and Hay Bale ML-based Identification, which includes training the evaluated model YOLOv11 and validating its performance using precision, recall, F1-score, and IoU metrics. The resulting *BaleUAVision* dataset includes flight paths, mission details, metadata CSVs, orthophotos, annotated data in multiple formats (COCO, CSV, JSON, YOLO, and segmentation masks), trained weights, and evaluation metrics such as precision, recall, F1-score, and IoU.

*BaleUAVision* is a comprehensive data set designed to advance precision agriculture through automated detection and counting of hay bales. It comprises 2,599 high-resolution RGB images (4056 × 3040 pixels) collected over 16 fields in Northern Greece (14 in Xanthi and 2 in Drama), covering a total area of 938,715 m^2^ (approximately 232 acres). The selected regions are representative hay-producing areas in Greece. Their climatic conditions, extensive plains, and strong livestock sector drive hay production, making them particularly relevant for capturing realistic bale appearances in UAV imagery. In addition to the raw images, the dataset provides thoroughly annotated versions—with semantic segmentation annotations delivered in multiple formats (COCO, CSV, JSON, YOLO, and segmentation masks) via Label Studio—and high-quality orthomosaics generated through an image stitching process. Flight parameters were varied (altitudes from 50 to 100 meters, speeds between 3.7 and 5 m/s, and overlap ratios from 55–80% frontal and 60–70% side) to ensure both high resolution and complete field coverage. Furthermore, manual hay bale counts for each field offer valuable ground truth for the development and evaluation of automated detection and counting algorithms (e.g.^24,25^). The complete workflow for developing the BaleUAVision dataset, from data acquisition to machine learning-based hay bale identification, is outlined in Fig. 1.

Organized into three distinct tiers, *BaleUAVision* is designed to maximize its utility across a range of research applications. Tier 1 provides the foundation with raw, high-resolution UAV imagery and processed orthomosaics produced via image stitching techniques. Tier 2 offers refined, polygon-based annotations where each hay bale is accurately delineated. Tier 3 includes comprehensive metadata—most notably, manual hay bale counts that serve as reliable ground truth for model training and evaluation. Overall, *BaleUAVision* addresses key limitations in existing remote sensing products and sets a new benchmark for agricultural and environmental data quality. By combining high-resolution imagery, diverse flight parameters, and a focused approach to hay bale detection and counting, the dataset delivers significant advantages for advancing precision agriculture, environmental monitoring, and the development of autonomous systems (e.g.^26,27^). In line with FAIR principles^28^, the complete dataset and its metadata are readily accessible^29^, while a meta-analysis on the data, including data statistics, insights, usage python scripts, and indicative examples are provided also^30^.

## Methods

**Fig. 2:**
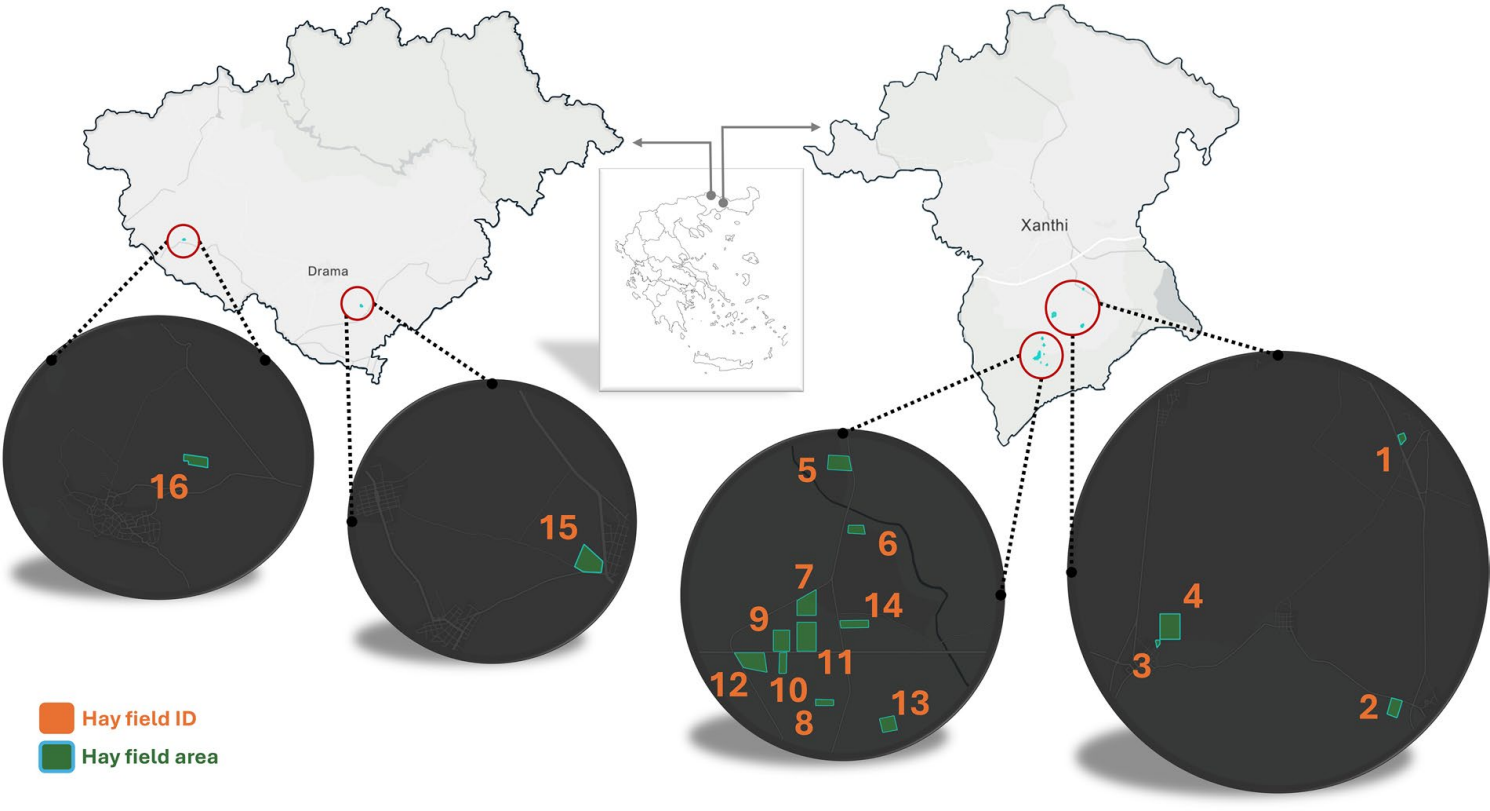
Geographic overview of the 16 hay fields included in the *BaleUAVision* dataset. The inset map in the center locates the Xanthi and Drama regions within Greece, while the magnified spherical views on either side detail the individual fields. Fields 1–14 lie in the Xanthi region, and fields 15–16 in the Drama region. Each orange label corresponds to the field ID, and the green polygons outline the approximate area of each hay field.

The creation of the *BaleUAVision* dataset followed a structured pipeline comprising two main stages: Data Acquisition and Data Processing. The Data Acquisition stage involved detailed mission design—including path planning and raw UAV data collection—conducted over agricultural fields in Northern Greece, specifically within the Xanthi and Drama regions (see Fig. 2 for a map of the flight locations). In the Data Processing stage, high-resolution orthomosaics were generated to provide an overarching view of the study areas, and to facilitate manual hay bale counting, with the resulting counts included in the metadata. However, data annotation was performed directly on each raw image, ensuring that each hay bale was precisely delineated without relying on the orthomosaic representations.

### Data Acquisition

To capture the required high-resolution imagery, UAV missions were planned and executed over 16 hay bale fields in Northern Greece, with 14 fields located in the Xanthi region and 2 in the Drama region. These regions were selected because they represent typical hay-producing areas of Northern Greece, with Xanthi characterized by extensive lowland plains and Drama by more heterogeneous, semi-mountainous landscapes. Both areas supply hay primarily to support regional livestock farming, and their differences in terrain, field geometries, and harvesting practices affect the spatial distribution, arrangement, and visibility of hay bales in UAV imagery. Including this variability in the dataset ensures that it captures diverse agricultural conditions, enhancing its value for training robust and generalizable detection models. Data acquisition was carried out, during summer of 2023, using the Mavic 2 Enterprise Dual, which features a high-resolution RGB sensor (4056 × 3040 pixels). Although the UAV is also equipped with a thermal camera, only the RGB sensor was used for this dataset. Flight planning was performed to design mission routes that comprehensively covered each agricultural parcel. Critical flight parameters including altitude, speed, and overlap ratios, were adjusted on a per-case basis, with the selections made to create a diverse set of cases across the subsets. Specifically, flights were conducted at altitudes of 50 m, 80 m, and 100 m, which are typical operational levels for agricultural UAVs. The flight speeds ranged between 3.7 m/s and 5 m/s, while frontal and side overlap ratios were maintained at 55–80% and 60–70%, respectively. During each mission, comprehensive metadata was recorded, including flight time, the number of images captured, Ground Sampling Distance (GSD, ranging from 1.53 to 3.06 cm/pixel), and the geographical area covered. This metadata (detailed in Table 1) provides essential context for subsequent data processing and machine learning applications. Additionally, every image in all subsets is geo-referenced, ensuring precise spatial localization for further analysis.

**Table 1:** Overview of UAV flight parameters and dataset metadata for *BaleUAVision*.

	Dataset ID (Study Site)															
	HB1	HB2	HB3	HB4	HB5	HB6	HB7	HB8	HB9	HB10	HB11	HB12	HB13	HB14	HB15	HB16
Altitude (*m*)	50	50	50	100	50	50	100	50	100	100	100	100	100	100	100	80
Takeoff Speed (*m*/*s*)	10	10	10	10	10	10	10	10	10	10	10	10	10	10	10	10
Speed (*m*/*s*)	3.7	3.7	3.7	5	3.7	3.7	5	3.7	5	5	5	5	5	5	5	5
Side Overlap (%)	70	70	70	70	70	70	70	60	70	70	70	70	60	65	60	60
Frontal Overlap (%)	80	80	80	80	80	80	80	70	80	80	80	55	65	70	65	70
GSD (*cm*/*pixel*)	1.53	1.53	1.53	3.06	1.53	1.53	3.06	1.53	3.06	3.06	3.06	3.06	3.06	3.06	3.06	2.45
Area (*m*^2^)	22,339	59,796	7,718	166,778	47,865	21,367	68,327	24,376	67,371	25,423	79,038	80,005	33,296	30,462	140,991	63,563
Flight Time	9 m3 s	18 m34 s	3 m55 s	19 m30 s	15 m23 s	8 m22 s	10 m24 s	6 m31 s	9 m17 s	3 m40 s	10 m52 s	11 m7 s	4 m7 s	4 m20 s	14 m25 s	8 m59 s
Photos	205	423	86	286	346	188	165	103	145	61	172	80	40	47	133	119
Orthophoto	Done	Done	Done	Done	Done	Done	Done	Done	Done	Done	Done	Done	Done	Done	Done	Done
Count	41+0	49+0	17+0	107+5	69+0	31+0	67+20	38+13	94+40	31+12	51+14	58+11	83+0	54+9	40+0	33+0
Flight Date	7/19/23	7/19/23	7/19/23	7/19/23	7/21/23	7/21/23	7/21/23	7/22/23	7/22/23	7/22/23	7/22/23	7/23/23	7/23/23	7/23/23	7/29/23	7/30/23
Region	Xanthi	Xanthi	Xanthi	Xanthi	Xanthi	Xanthi	Xanthi	Xanthi	Xanthi	Xanthi	Xanthi	Xanthi	Xanthi	Xanthi	Drama	Drama

^*^HB stands for Hay Bales

^*^Latitude and longitude details are available in the metadata file *Dataset Description.csv*

^***^Count represents the total number of hay bales manually identified by a human observer in the orthophotos, both within the primary field and in the surrounding area visible in the images (field + surrounding area).

### Data Processing

**Fig. 3:**
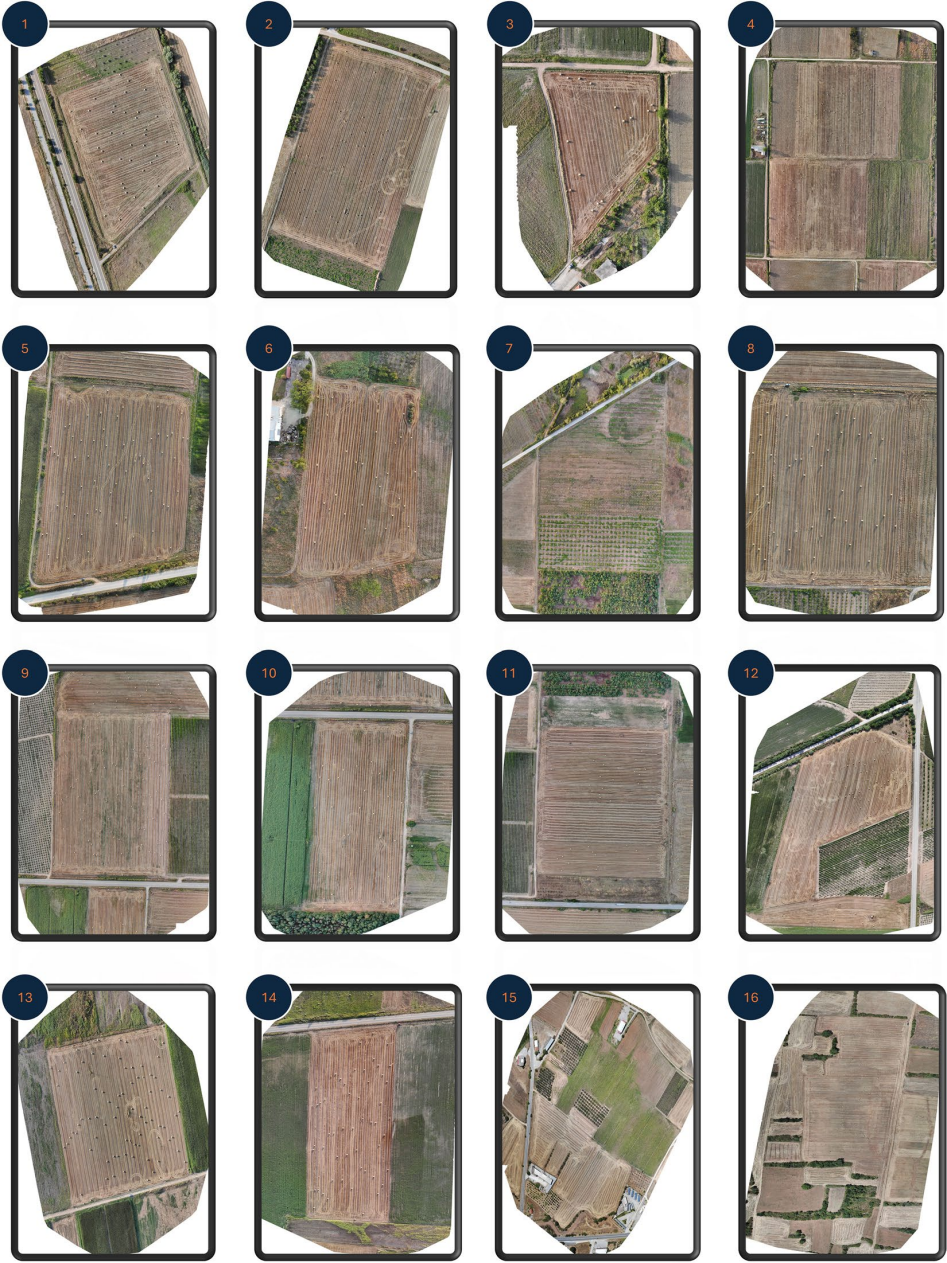
Orthophotos of the 16 hay bale fields in the *BaleUAVision* dataset. The numbering of each field is aligned with the one depicted in Fig. 2. Each orthophoto was generated by stitching multiple UAV-captured images using *WebODM*, providing a broad overview of each field. These orthophotos were used for manual hay bale counting, as reflected in Table 1.

After data acquisition, a thorough data processing phase was undertaken to ensure the quality and versatility of the dataset. Initially, all captured images were manually reviewed to identify and discard a small number of images containing sensitive data or exhibiting quality issues such as motion blur or sensor errors. Data processing then followed two parallel streams. In the first stream, images from each field were processed using the photogrammetry software *WebODM* (https://www.opendronemap.org/webodm/) to generate high-resolution orthomosaics. These orthophotos provide an overarching view of each study area (Fig. 3) and were used for manual hay bale counting, as detailed in Table 1.

**Fig. 4:**
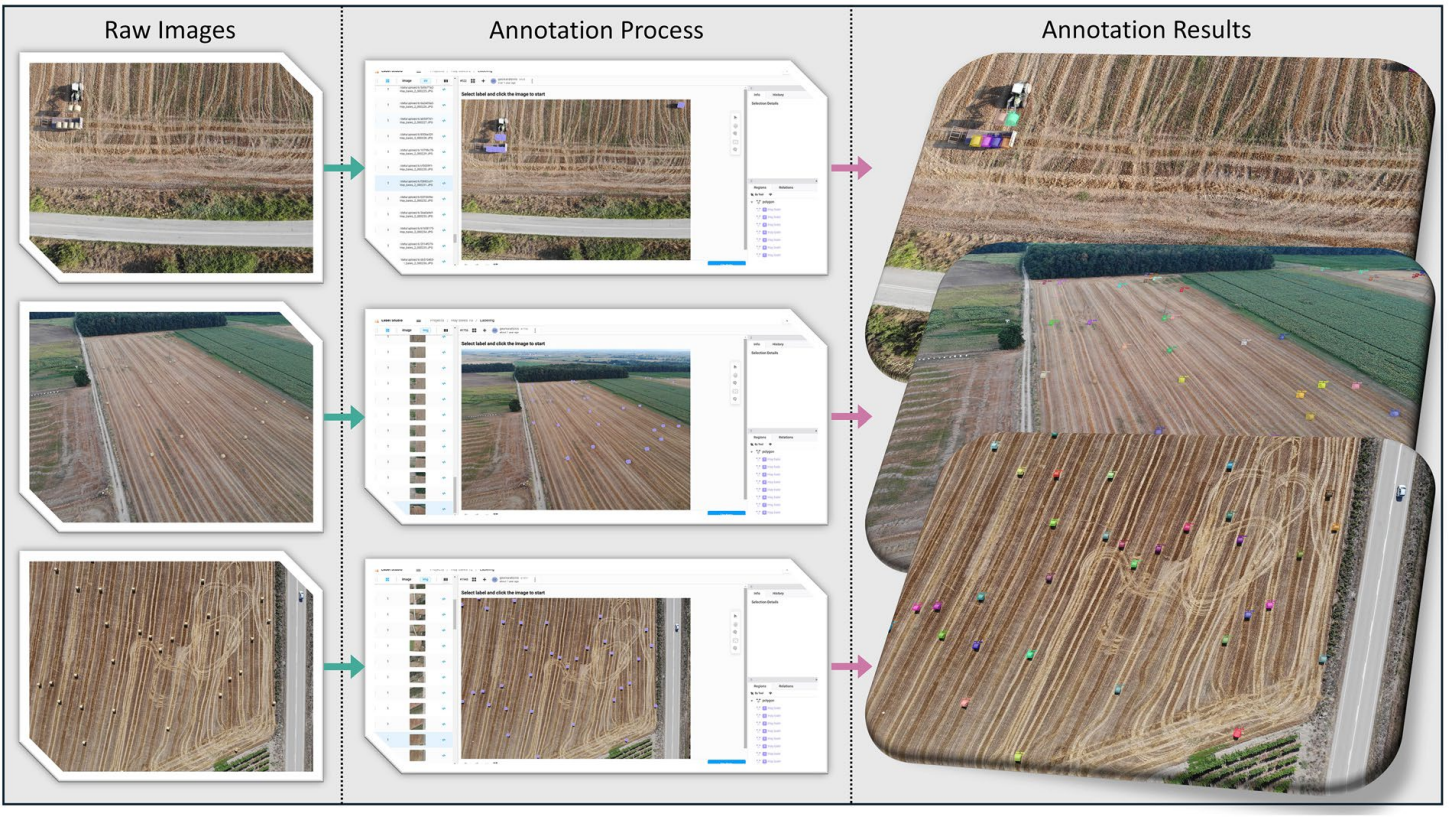
Illustration of the annotation workflow used in *BaleUAVision*. The raw UAV images (left) are loaded into the Label Studio interface (center), where each hay bale was manually delineated using polygon-based segmentations. The final annotated images (right) show color-coded polygons outlining each bale, providing high-precision segmentation data for downstream machine learning and deep learning tasks.

In the second stream, extensive efforts were dedicated to data annotation. All annotations were performed by a single annotator with expertise in UAV agricultural imagery. To ensure consistency, we adopted a structured annotation protocol consisting of the following steps: (1) each bale was delineated with a polygon along its outer contour; (2) partially visible bales (e.g. at image borders) were included; (3) bales that appeared close together or partly occluded were annotated as distinct instances; (4) shadows were not annotated, with labels restricted to the physical bale objects; and (5) a second review pass was conducted over all images to minimize omissions or inconsistencies. This procedure ensured accurate and uniform annotations throughout the dataset. A general overview of the annotation process is provided in Fig. 4. Using the open-source tool *Label Studio* (https://labelstud.io/), each hay bale in the raw images was manually delineated using polygon-based annotations. This approach was chosen over bounding boxes to capture the precise shape and size of each bale, thereby enhancing the dataset’s versatility for both classification and segmentation tasks—even though it was labor-intensive. To further maximize the dataset’s applicability, annotations were exported in several standard formats, including COCO, CSV, JSON, YOLO, and as segmentation masks, thereby supporting a wide range of machine learning workflows. A systematic file naming convention was implemented during the export process, with unique prefixes added to each image name to ensure consistency between the raw images and their corresponding annotations. The directory structure, as will be detailed in the next Section, was designed to facilitate easy navigation and usage.

## Data Records

**Fig. 5:**
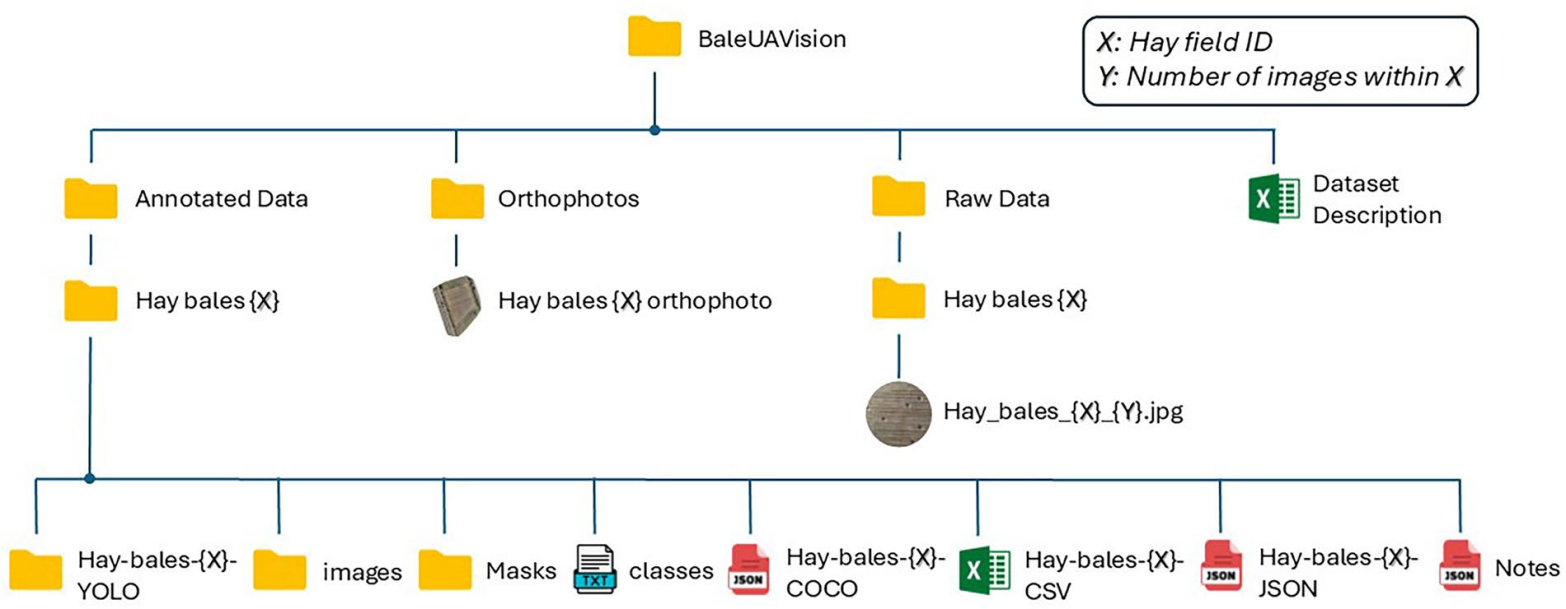
Schematic overview of the *BaleUAVision* dataset folder structure. Each Hay bales {X} subfolder contains the raw and annotated data specific to one flight mission. The Codes folder includes Python scripts for visualizing annotations, while Orthophotos provides high-resolution orthomosaic images.

All data comprising the *BaleUAVision* dataset are publicly available in the Zenodo repository^29^, organized in a structured hierarchy to facilitate straightforward navigation. A schematic of the folder structure is provided in Fig. 5, highlighting the primary components.

### Environmental Conditions During Data Acquisition

All UAV missions were conducted during the summer of 2023 in the regions of Xanthi and Drama, Greece, under stable weather with clear skies and no precipitation, ensuring high image clarity and consistent visibility across all flights. Each mission was executed under similar wind conditions (below 3 m/s) and moderate ambient temperatures typical of Mediterranean summer evenings. To quantify illumination conditions, solar elevation and azimuth angles were computed for every flight using the recorded date, time, and geographic coordinates. The analysis revealed a broad range of lighting conditions: solar elevations varied from as low as 2–20^°^ during late-evening flights in Xanthi to 30–40^°^ in mid-afternoon and up to 60–66^°^ around midday during the Drama campaigns. This variation introduced natural differences in the illumination geometry, which affected shadow length, contrast, and color tone across the data set.

The presence of both high and low solar elevations provides a realistic range of lighting conditions that can influence model generalization, particularly for outdoor detection tasks where shadow behavior and reflectance patterns vary with time of day. However, it should be noted that all data were acquired under clear-sky conditions and the dataset does not include overcast, cloudy, or dawn/dusk imagery. Overall, the combination of stable meteorological conditions with naturally varying solar illumination enhances dataset uniformity while still offering illumination diversity sufficient to test detection performance under different lighting geometries. Finally, the dataset does not include multiple acquisition dates per field, as each site was surveyed once under stable post-harvest conditions.

### Metadata

**Fig. 6:**
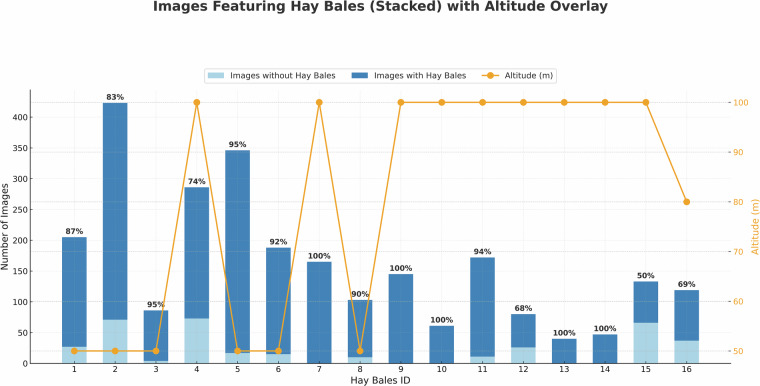
Overview of the *BaleUAVision* dataset, detailing the total number of images, the percentage of images containing annotations for each hay bale subset, and the corresponding flight altitudes at which the data were collected.

A comprehensive CSV file, *Dataset Description.csv*, is provided at the root level. This file contains essential flight parameters (e.g. altitude, speed, overlap ratios), Ground Sampling Distance (GSD), latitude and longitude of the flight polygon for every case, flight date, region, total number of images, and manually counted hay bales for each field. These metadata offer critical context for understanding both the acquisition conditions and potential downstream analyses, while also the exact GPS coordinates of the hay bay fields. A general overview of the *BaleUAVision* dataset is depicted in Fig. 6, presenting the number of images and the percentage of annotated images per hay bale subset, as well as the flight altitude for each case.

### Raw Data

All original UAV-captured images are stored in the Raw Data folder. Each subfolder (Hay bales {X}) corresponds to a single flight mission over one of the 16 fields. Note that {X} stands for the hay field ID. Images are geo-referenced and retain their full resolution of 4056  × 3040 pixels.

### Annotations

Manually created polygon-based annotations are positioned within the Annotated Data folder, organized by field case (e.g. Hay bales {X}). Each subfolder contains:

- images: Includes the annotated images with unique file-name prefixes generated by the labeling software. For example in the Hay bales {1} case folder, the prefix "ff4026a8-" is added to the original name Hay_bales_1_00057.JPG. This is a standard property on how Label Studio handles the export process, where adding a unique prefix ensures that each image file name is distinct, which helps in managing and referencing the images correctly in the dataset. Practically a unique prefix is added to every image for every sub-set of the annotated part in accordance with the original raw dataset (Raw Data). Note also that Label Studio often uses paths to refer to the location where the files are stored. Thus, the users may notice for example a path like /data/upload/5/089c0055-Hay_bales_1_0001.JPG in the CSV version. In this case, 089c0055-Hay_bales_1_0001.JPG is the file name. Therefore, users can manipulate the strings to extract only the file names in their code in the sense of having the same file name that corresponds to the image in “images” folder excluding the path string and keeping only the filename.
- Multiple annotation formats: COCO, CSV, JSON, YOLO, and segmentation masks, ensuring compatibility with a wide range of computer vision frameworks.
- classes and notes files with auxiliary information for certain annotation formats.

### Orthophotos and Supporting Files

High-resolution orthomosaic images, generated using photogrammetry software (WebODM), are placed in the Orthophotos folder. The orthomosaic mappings are the result of a classic image stitching process that encompasses all individual UAV-captured images for each hay field to produce the unified representation panorama. These orthophotos were employed for manual hay bale counting, as reflected in the metadata CSV file *Dataset Description.csv*. The geometric quality of the orthomosaics was evaluated using the quality assessment reports automatically generated by WebODM across representative missions covering the full range of flight altitudes (50 to 100 m). These reports provided quantitative metrics on spatial resolution, reprojection accuracy, and georeferencing consistency, confirming the strong geometric integrity of the dataset. According to the WebODM reports, the average ground sampling distance (GSD) ranged between 1.6 cm/pixel and 3.4 cm/pixel, values that align closely with the expected spatial resolutions derived from the planned flight altitudes and camera specifications. This agreement demonstrates stable scaling across the mosaics, with negligible deviation from the designed acquisition parameters. The average reprojection error remained below 0.65 pixels, indicating excellent tie-point alignment and minimal internal distortion, while the 3D RMS error ranged from 0.04 m to 0.06 m, reflecting high geometric stability of the reconstructed surfaces.

The GPS-based georeferencing RMS positional errors were found to range from 2.8 m to 5.6 m, corresponding to horizontal (CE90) accuracies of 1.6 to 2.3 m and vertical (LE90) accuracies of 4.8 to 9 m. These values are consistent with expected precision for consumer-grade GNSS systems without ground control points. Despite the absence of GCPs, the combination of high image overlaps (70–80% frontal, 60–70% lateral) and accurate camera calibration ensured strong relative alignment and reliable local geometry throughout all reconstructions. Overall, both the quantitative WebODM metrics and the visual inspection of the orthomosaics and structural edges confirm that they are geometrically accurate, internally consistent, and exhibit uniform spatial scale. These properties make the dataset suitable for centimeter-level annotation, manual counting, and object-detection applications among other usages.

## Technical Validation

To validate the technical soundness of our dataset, we employed an object detection task as a benchmark, focusing on two key scenarios: location invariance and altitude invariance for hay bales detection. To ensure a robust evaluation, we utilized YOLOv11, a widely adopted detection model known for its high performance, ease of use, and strong generalization across diverse detection tasks. Its efficiency and reliability make it an ideal choice for assessing our dataset’s capabilities in real-world UAV applications.

### Implementation Details

For our implementation, we used YOLOv11s with an input image size of 640 × 480, following the default configurations from the official repository. We experimented with different optimizers and learning rates, but the auto-scaler provided in the YOLOv11 repository consistently yielded the best results. The model was trained for 100 epochs, with minimal hyperparameter tuning, as our primary goal was not to maximize performance but rather to demonstrate the dataset’s potential in a realistic object detection setup.

### Validation Scenarios

To evaluate the dataset, we designed two validation scenarios that reflect common sources of variation in UAV-based monitoring: (i) location invariance, to assess whether models trained in one geographic region can generalize to another; and (ii) altitude invariance, to examine the effect of different ground sampling distances on detection robustness. These scenarios were selected because they represent realistic operational challenges for UAV imagery, where fields vary geographically and flights are performed at different altitudes. The following subsections present the experimental setup and results for each case. In addition, a discussion subsection examines the effect of flight parameters on data quality, and a comparison with foundation models is provided to demonstrate the advantages of training on domain-specific agricultural data.

#### Scenario 1 - Location Invariance

In the location invariance scenario, we structured the dataset based on geographical regions, training on images from locations in Xanthi(HB1-HB14) and validating on images from Drama(HB15,HB16). This split ensures zero overlap between training and validation sets while maintaining some environmental similarities, as both regions belong to Northern Greece. However, minor variations in terrain and environmental features introduce natural diversity, making this a meaningful test for location invariance. While we do not claim that our model is fully location-invariant, our results indicate that training on our dataset enables generalization across moderate environmental shifts, highlighting its potential for practical applications.

Table 2 presents the model’s performance on the location study. The results are extracted from the epoch with best mAP@0.5:0.95. These results highlight the strong generalization capabilities of the model across geographically distinct areas. The high precision (0.99) indicates a very low false positive rate, meaning the model rarely misclassifies non-bale objects as bales. Additionally, the recall score of 0.94 suggests that the majority of actual bale instances were successfully detected, despite the shift in location. The slight drop in mAP@0.5:0.95 compared to mAP@0.5 (0.69 vs 0.98) is expected, as the stricter IoU thresholds in mAP@0.5:0.95 penalize minor localization inaccuracies. Nevertheless, the overall performance is robust, confirming that the dataset provides sufficient diversity for the model to handle moderate changes in environment and terrain.

**Table 2:** Detection performance for Scenario 1 (Location Invariance).

Metric	Value
mAP@0.5	0.98
mAP@0.5:0.95	0.69
Recall	0.94
Precision	0.99

Again, we do not claim complete location invariance, as environmental and visual differences between regions may still introduce edge cases that challenge the model. However, the strong results presented here suggest a meaningful degree of generalization. The model’s ability to maintain high recall and precision across unseen geographic locations indicates that it captures features that are robust to moderate spatial variation. This reinforces the potential of our dataset for broader deployment in UAV-based monitoring tasks, particularly in agricultural settings where field conditions vary across locations.

#### Scenario 2 - Altitude Invariance

The altitude invariance scenario addresses a common challenge in UAV-based datasets—altitude drift. In real-world operations, UAV flight constraints often lead to variations in altitude, which can significantly impact model performance. To assess the model’s adaptability to such changes, we conducted missions at 50 m, 80 m, and 100 m, varying per location. Initially, two experimental setups were designed:

- Training at 50 m and 80 m (1,470 images) and testing at 100 m (1,129 images).
- Training at 100 m (1,129 images) and testing at 50 m and 80 m (1,470 images).

These experiments help quantify the model’s robustness in handling altitude discrepancies, providing valuable insights into its real-world applicability. By leveraging YOLOv11s, we ensure that our evaluations reflect state-of-the-art detection performance. Table 3 showcases the results of Scenario 2a, where the model was trained at lower altitudes (50 m and 80 m) and tested at 100 m. The model achieved a high mAP@0.5 of 0.95 and a respectable mAP@0.5:0.95 of 0.67. Precision remained strong at 0.97, indicating confident and accurate detections, while recall at 0.90 shows that the model effectively identified the majority of bale instances even with increased object scale compression due to altitude.

**Table 3:** Detection performance for Scenario 2a (Altitude Invariance: 50 m/80 m  → 100 m).

Metric	Value
mAP@0.5	0.95
mAP@0.5:0.95	0.67
Recall	0.90
Precision	0.97

In contrast in Table 4, Scenario 2b (training at 100 m and testing at 50 m and 80 m) showed a more pronounced performance drop, particularly in mAP@0.5:0.95 (0.55) and recall (0.80). This suggests that models trained at higher altitudes may struggle to generalize to lower-altitude imagery, possibly due to the increased object resolution and detail that were underrepresented in training. It was evident during training that the model struggled in the validation set as we observed significant variance in validation metrics across epochs.

**Table 4:** Detection performance for Scenario 2b (Altitude Invariance: 100 m  → 50 m/80 m).

Metric	Value
mAP@0.5	0.93
mAP@0.5:0.95	0.55
Recall	0.80
Precision	0.89

While Scenario 2a (lower to higher altitude) demonstrated strong generalization, Scenario 2b (higher to lower) revealed a noticeable performance drop, particularly in recall and mAP@0.5:0.95. This suggested that models trained solely at higher altitudes may struggle to adapt to the greater detail and scale variations present in lower-altitude images. To investigate whether even minimal low-altitude exposure could mitigate this issue, we introduced a third setup:

c. Training at 100 m with the addition of a hay field (HB1) captured at 50 m (1,334 images), then testing at 50 m and 80 m (1,265 images).

**Fig. 7:**
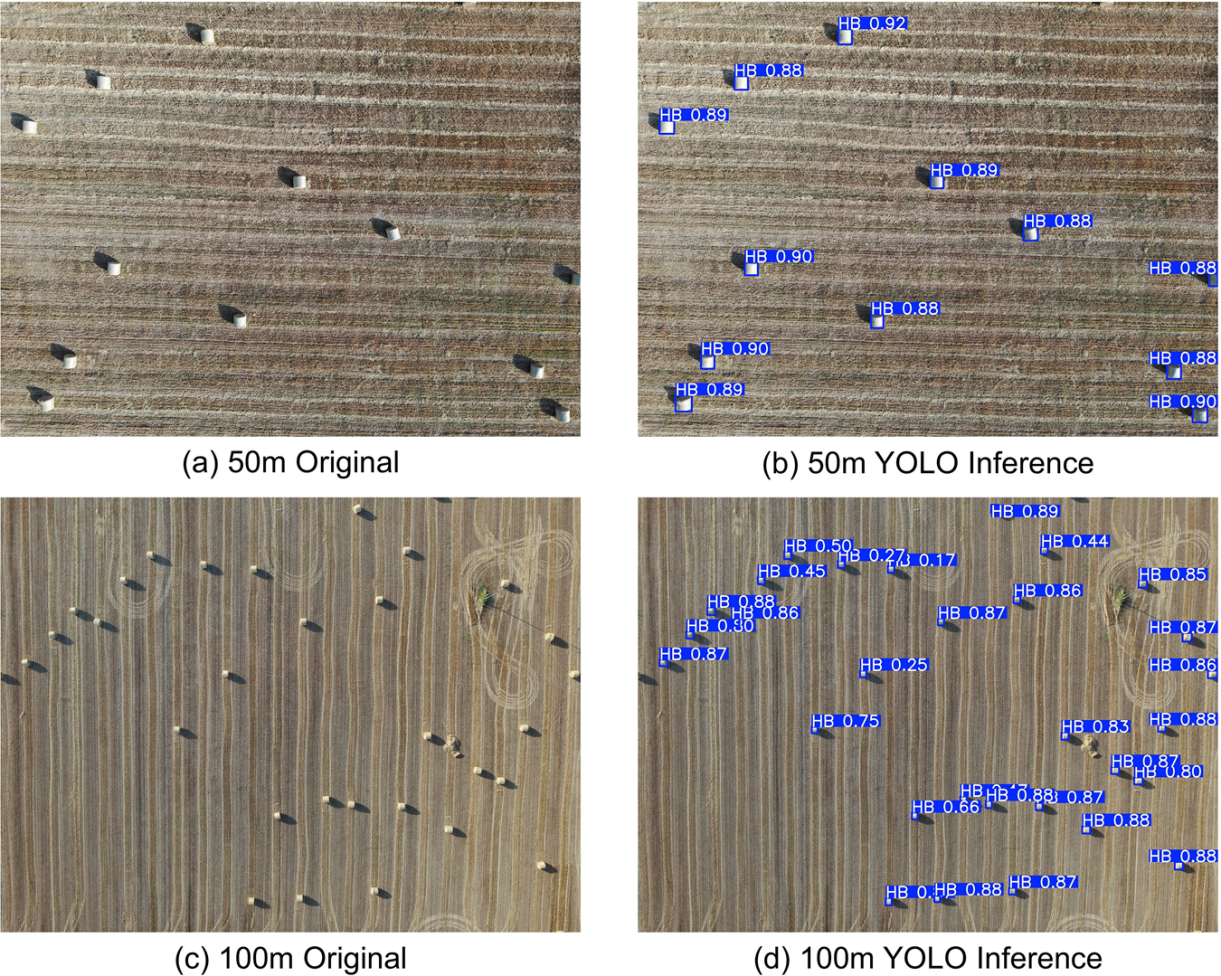
YOLOv11s inference results at 50 m and 100 m altitude on validation set.

This third case aimed to evaluate whether a small, targeted injection of low-altitude data could improve downward generalization. Interestingly, Scenario 2c demonstrated a notable performance uplift compared to Scenario 2b as depicted in Table 5. The mAP@0.5 increased to 0.97 and mAP@0.5:0.95 rose to 0.74, accompanied by higher recall (0.94) and precision (0.98). These results suggest that even limited exposure to low-altitude imagery can significantly enhance the model’s ability to generalize downward in altitude. While full altitude diversity remains ideal, these findings highlight the value of strategic sampling, where incorporating just a small amount of representative data can lead to substantial gains in robustness and reliability across altitude variations. Two indicative examples are depicted in Fig. 7 for 50 m and 100 m images from the validation setup along with the model’s confidence. These findings revealed that altitude variations do impact detection performance, with training at lower or mixed altitudes offering better generalization. Including representative altitude diversity appears to be a key factor in achieving robust UAV-based object detection under real-world constraints.

**Table 5:** Detection performance for Scenario 2c (Altitude Invariance: 100 m + single hay field 50 m)  → 50 m/80 m).

Metric	Value
mAP@0.5	0.97
mAP@0.5:0.95	0.74
Recall	0.94
Precision	0.98

The results of our object detection experiments underscore the technical soundness and practical value of the proposed dataset. The location invariance scenario demonstrated strong generalization across geographically distinct regions, while the altitude invariance experiments revealed the importance of incorporating diverse altitude perspectives during training. Notably, even limited inclusion of low-altitude data substantially improved the model’s robustness, emphasizing the value of strategic data sampling. Overall, these findings validate the dataset’s applicability in real-world UAV operations and highlight its potential as a reliable resource for training high-performance object detection models in agricultural and remote sensing contexts.

#### Effect of Flight Parameters on Data Quality

Beyond the detection results, it is important to briefly reflect on how flight parameters (altitude, speed, and overlap) influenced annotation quality and dataset balance. Altitude determined the ground sampling distance (GSD), with lower flights (50–80 m,  ≈1.5–2.5 cm/pixel) yielding finer object detail that facilitated more precise polygon delineation, while higher flights (100 m,  ≈3 cm/pixel) provided broader coverage but occasionally required greater care during annotation. Overlap ratios (frontal 55–80%, side 60–70%) primarily affected orthomosaic reconstruction; higher overlaps generally produced cleaner stitched images, but no systematic inconsistencies were observed in raw image annotations across different overlap levels. UAV speed (3.7–5 m/s) had negligible impact on annotation quality, as motion blur was screened out during manual review. In terms of model performance, the altitude invariance experiments (Scenario 2 - Altitude Invariance) indirectly capture these effects, as coarser GSD at higher altitudes contributed to reduced generalization when training data lacked lower-altitude examples. Overall, while overlap and GSD shaped the annotation workflow, the dataset remains consistent across conditions and supports robust model development. In relation to Scenario 1 - Location Invariance, flight parameters played a limited role. The primary source of variation in that case stemmed from differences in terrain, vegetation background, and lighting conditions between Xanthi and Drama. These factors represent a complementary source of variability to altitude, overlap, and speed, emphasizing that the dataset captures both geographic and flight-related sources of diversity that are relevant for UAV-based monitoring.

#### Comparison with Foundation Models and Domain-Specific Relevance

To contextualize our results within current general-purpose vision research, we compared a YOLOv11 detector trained on *BaleUAVision* against SAM2^31^, OWL-V2^32^, and an OWL-SAM pipeline on the Location Invariance scenario (train: Xanthi HB1-HB14; validate: Drama HB15-HB16). Foundation models are strong zero-/few-shot learners, but agricultural UAV imagery presents challenging characteristics (small object scale, repetitive textures, background clutter). To ensure fairness, we tuned model-specific thresholds and post-processing and filtered detections with implausible bale sizes.

Table 6 shows that the model trained on *BaleUAVision* consistently outperforms general-purpose baselines. The OWL-SAM pipeline is the strongest among them, yet our model remains ahead in both detection accuracy and localization quality. OWL-V2 trails further, and SAM2 performs weakest in this setting. Notably, all foundation models exhibit a marked drop from mAP@0.5 to mAP@0.5:0.95, indicating difficulties with precise box placement on small, texture-rich hay bales amid cluttered backgrounds, an area where domain-specific training proves especially beneficial.

**Table 6:** Comparison with foundation models on Scenario 1 (Location Invariance).

Metric	Ours	SAM2	OWLv2	OWLv2-SAM
Recall	0.94	0.83	0.78	0.92
Precision	0.99	0.17	0.35	0.60
mAP@0.5	0.98	0.07	0.62	0.90
mAP@0.5:0.95	0.69	0.03	0.27	0.34

The comparison with foundation models highlights three important observations about the role of *BaleUAVision* within this context. *(i) Domain-specific supervision*. Training on *BaleUAVision* substantially improves both detection accuracy and localization precision compared to zero- or few-shot foundation models, particularly for small, cluttered agricultural targets. *(ii) Complementarity*. Rather than competing with general-purpose vision systems, *BaleUAVision* complements them by providing high-quality, domain-relevant data that enables fine-tuning and stable performance under varying geographic and environmental conditions. *(iii) Deployment practicality*. In real-world UAV applications with limited onboard computation, a YOLOv11 model trained on *BaleUAVision* offers a superior balance between accuracy and efficiency compared to SAM- or OWL-based pipelines, making it more suitable for edge deployment.

## Usage Notes

### Software Requirements

- Label Studio: Used for creating and exporting annotations. Researchers can import the provided annotation projects to modify or verify annotations.
- WebODM: Employed for orthomosaic generation. This is useful for recreating orthophotos or processing additional UAV imagery with similar parameters.
- Dependencies: Ensure Python environments include libraries specified in the GitHub README (e.g. Matplotlib, Pandas) to run the provided scripts effectively.

### Recommended Best Practices

- To maximize the value of *BaleUAVision* for generalization across different altitudes and geographical locations, researchers should incorporate diverse training subsets provided within the dataset. The technical validation section (Scenario 2) offers insights into strategic sampling for altitude invariance.
- For optimal results and replication, users should follow the provided metadata closely, particularly with respect to flight parameters and Ground Sampling Distance (GSD), to ensure accurate modeling and interpretation.
- Geo-referencing: Leverage the GPS metadata in raw images for spatial analyses, such as mapping hay bale locations or integrating with GIS platforms.
- Researchers are encouraged to explore the GitHub repository for additional data statistics, insights, and code examples to fully leverage the dataset’s capabilities.

### Potential Applications Beyond Hay Bale Detection

While *BaleUAVision* was initially designed to enable automated hay bale detection and counting, its high-resolution UAV imagery, detailed annotations, and georeferenced orthophotos make it a valuable resource for a wide range of related research activities in precision agriculture and computer vision. The dataset’s structure and metadata enable its use not only for object-level analysis but also for broader spatial and operational studies.

- **Object detection and counting**. The dataset provides a high-quality benchmark for developing, training, and evaluating algorithms for object detection and instance segmentation in aerial imagery, including small and repetitive objects typical of agricultural environments.
- **Agricultural field assessment**. The imagery and orthophotos can support analyses of field texture, residue distribution, or post-harvest surface patterns, offering insights into field conditions and management practices.
- **Resource allocation and logistics**. By analyzing bale spatial distribution patterns, the dataset can inform logistics and operational planning, such as optimizing bale collection routes, storage strategies, or transport scheduling in real-world agricultural workflows.
- **Training and benchmarking of vision models**. *BaleUAVision* can serve as a benchmark dataset for developing and evaluating computer vision models for object detection or segmentation in agricultural UAV imagery, particularly where small and repetitive objects are present.
- **Simulation Scenarios for Robotics**. The dataset can be used in simulation environments for testing perception and navigation modules of unmanned ground or aerial vehicles, particularly for bale collection or monitoring tasks.
- **Adaptation of general-purpose models**. The polygon-based annotations and diverse flight conditions make the dataset suitable for evaluating or fine-tuning general-purpose segmentation models (e.g. SAM, SAM2 or OWL-V2) on agricultural UAV imagery.

These applications demonstrate that *BaleUAVision* can contribute not only to hay bale detection but also to broader research efforts in data-driven field monitoring, logistics optimization, and autonomous agricultural systems.

### Dataset Limitations

While *BaleUAVision* offers a high-quality and diverse collection of UAV-captured imagery, certain scope-related limitations should be acknowledged. All missions were conducted during summer 2023 under stable weather and clear-sky conditions, which coincide with the hay harvesting period in Northern Greece. This ensured consistent visibility and representative field appearances but excluded other seasonal or meteorological variations such as cloudy, rainy, or off-season conditions. The dataset currently covers two agricultural regions in Northern Greece (Xanthi and Drama), representing typical hay production areas but not the full range of geographic or climatic diversity found elsewhere. These factors reflect the dataset’s focus on providing clear, high-resolution imagery for object detection and counting, while leaving room for future extensions that incorporate multi-season, multi-modal, or broader geographic data.

## Data Availability

All data comprising the *BaleUAVision* dataset are publicly available in the Zenodo repository^29^.

## Code availability

All related code, including data statistics, insights, usage python scripts, and indicative examples are provided at the living page of the project^30^.
